# Recent trends in publication of basic science and clinical research by United States investigators in anesthesia journals

**DOI:** 10.1186/1471-2253-12-5

**Published:** 2012-03-22

**Authors:** Paul S Pagel, Judith A Hudetz

**Affiliations:** 1 Professor of Anesthesiology, the Clement J. Zablocki Veterans Affairs Medical Center, 5000 West National Avenue, Milwaukee, WI 53295, USA; 2 Assistant Professor of Anesthesiology, the Clement J. Zablocki Veterans Affairs Medical Center, 5000 West National Avenue, Milwaukee, WI 53295, USA; 3 Clement J. Zablocki Veterans Affairs Medical Center, Anesthesia Service, 5000, W. National Avenue, Milwaukee, WI 53295, USA

**Keywords:** Anesthesia journals, Bibliometrics, Research, Scholarship, Scientific publication

## Abstract

**Background:**

United States anesthesia research production declined sharply from 1980-2005. Whether this trend has continued despite recent calls to improve output is unknown. We conducted an observational internet analysis to quantify American basic science and clinical anesthesia research output in 14 anesthesia journals with impact factors greater than one at three-year intervals during the past decade.

**Results:**

American investigators published 1,486 (21.7%) of the total of 6,845 research articles identified in anesthesia journals in 2001, 2004, 2007, and 2010. Approximately two-thirds of all US articles were published in *Anesthesiology *and *Anesthesia and Analgesia*. There was a significant correlation (r^2 ^= 0.316; P = 0.036) between the number of articles published by American authors in each anesthesia journal and the corresponding journal's impact factor in 2010. Significantly (P < 0.05; Pearson's Chi-square) fewer basic science articles were published in 2007 and 2010 compared with 2001. US clinical research output also declined in 2007 (201; 15.7%) compared with 2001 (266; 19.1%) and 2004, but an increase occurred in 2010 (279; 21.8%, P < 0.05 versus 2007).

**Conclusions:**

The results indicate that US anesthesia research output continued to decrease from 2001 to 2007. An increase in clinical but not basic science research was observed in 2010 compared with 2007, suggesting that a modest recovery in clinical research production may have begun.

## Background

In 2003, Szokol *et al *reported that the percentage of total basic science and clinical research papers published by American authors in *Anesthesiology, Anesthesia and Analgesia*, and *Pain *had decreased substantially between 1980 and 2000 [1]. These data reflected earlier observations of declining United States (US) production in other medical specialties [2-4]. Greater clinical commitments and proportionally less research activity because of personnel shortages, expanding services within and outside the operating room, and decreasing reimbursement most likely played important roles in the declining number of US papers, as did a progressive increase in the quality of research submissions from other countries to these and other anesthesia journals [1]. Lack of effective senior faculty research mentoring and a consequent inability of new investigators to successfully earn increasingly competitive US National Institutes of Health (NIH) funds were also identified as key factors in the decline of American anesthesia research [5]. New strategies to revive research were proposed in a number of editorials written by experts in anesthesia research approximately five years ago [5-9]. Whether US research output has increased since in response to or has continued to decrease despite these and other attempts to improve the number or quality of papers produced is unknown. We examined basic science and clinical research articles in anesthesia journals at selected intervals in the past decade to quantify recent trends in US research production.

## Results and discussion

A total of 6,845 basic science and clinical research articles were identified, of which American authors published 1,486 (21.7%). The total number of articles published in all 14 journals decreased modestly from 2001 (1,817) to 2010 (1,580); a decline in the number of articles appearing in *Anesthesiology *and *Anesthesia and Analgesia *was primarily responsible for this observation (Table 1). Approximately two-thirds of all US articles were published in *Anesthesiology *and *Anesthesia and Analgesia*, whereas American authors infrequently published their work in *Anaesthesia, Anaesthesia and Intensive Care*, and the *European Journal of Anaesthesiology*. Nevertheless, there was a relatively weak, but statistically significant (P = 0.036), correlation (r^2 ^= 0.316) between the number (n) of articles published by American authors in each anaesthesia journal and the corresponding journal's impact factor in 2010.

**Table 1:** Research articles for each journal

Journal	2010 IF	2001 US/Total (%)	2004 US/Total (%)	2007 US/Total (%)	2010 US/Total (%)	Total US/Total (%)
*Anesthesiology *(US)	5.486	113/295 (38.3)	137/311 (44.1)	72/197 (36.6)	86/205 (42.0)	408/1008 (40.5)
Basic Science		68/145 (46.9)	79/153 (51.6)	42/87 (48.3)	43/90 (47.8)	232/475 (48.8)
Clinical		45/150 (30.0)	58/158 (36.7)	30/110 (27.3)	43/115 (37.4)	176/533 (33.0)
*British Journal of Anaesthesia *(UK)	4.224	9/196 (4.6)	16/173 (9.3)	3/184 (1.6)	15/158 (9.5)	43/711 (6.1)
Basic Science		2/33 (6.1)	4/39 (10.3)	1/25 (4.0)	3/37 (8.1)	10/134 (7.5)
Clinical		7/163 (4.3)	12/134 (9.0)	2/159 (1.3)	12/121 (9.9)	33/577 (5.7)
*Anesthesia and Analgesia *(US)	3.274	161/451 (35.7)	151/470 (32.1)	124/371 (33.4)	122/308 (39.6)	558/1600 (34.9)
Basic Science		53/131 (40.5)	55/140 (39.3)	47/144 (32.6)	25/87 (28.7)	180/502 (35.9)
Clinical		108/320 (33.8)	96/330 (29.1)	77/227 (33.9)	97/221 (43.9)	378/1098 (34.4)
*Anaesthesia *(UK)	3.008	0/141 (0.0)	2/123 (1.6)	0/122 (0.0)	3/106 (2.8)	5/492 (1.0)
Basic Science		0/19 (0.0)	0/16 (0.0)	0/8 (0.0)	0/3 (0.0)	0/46 (0.0)
Clinical		0/121 (0.0)	2/107 (1.9)	0/114 (0.0)	3/103 (2.9)	5/446 (1.1)
*Regional Anesthesia and Pain Medicine *(US)	2.807	19/61 (31.1)	9/36 (25.0)	12/49 (24.5)	22/55 (40.0)	62/201 (30.9)
Basic Science		2/8 (25.0)	3/8 (37.5)	2/12 (16.7)	6/14 (42.9)	13/42 (31.0)
Clinical		17/53 (32.1)	6/28 (21.4)	10/37 (27.0)	16/41 (39.0)	49/159 (30.8)
*Journal of Neurosurgical Anesthesiology *(US)	2.205	10/33 (30.3)	12/27 (44.4)	5/33 (15.2)	11/47 (23.4)	38/140 (27.1)
Basic Science		5/12 (41.7)	1/3 (33.3)	1/6 (16.7)	3/8 (37.5)	10/29 (34.5)
Clinical		5/21 (23.8)	11/24 (45.8)	4/27 (14.8)	8/39 (20.5)	28/111 (25.2)
*Acta Anaesthesiologica Scandinavica *(Europe)	2.196	9/148 (6.1)	5/150 (3.3)	4/147 (2.7)	3/131 (2.3)	21/576 (3.7)
Basic Science		7/35 (20.0)	1/31 (3.2)	0/27 (0.0)	0/21 (0.0)	8/114 (7.0)
Clinical		2/113 (1.8)	4/119 (3.4)	4/120 (3.3)	3/110 (2.7)	13/462 (2.8)
*Canadian Journal of Anesthesia *(Canada)	2.180	13/129 (10.1)	13/109 (11.9)	8/63 (12.7)	8/65 (12.3)	42/366 (11.5)
Basic Science		4/18 (22.2)	3/16 (18.8)	3/9 (33.3)	1/8 (12.5)	11/51 (21.6)
Clinical		9/111 (8.1)	10/93 (10.8)	5/54 (9.3)	7/57 (12.3)	31/315 (9.8)
*Pediatric Anesthesia *(UK)	2.173	11/69 (15.9)	16/78 (20.5)	17/106 (16.0)	32/101 (31.7)	76/354 (21.5)
Basic Science		0/0 (0.0)	1/1 (100.0)	0/4 (0.0)	0/0 (0.0)	1/5 (20.0)
Clinical		11/69 (15.9)	15/77 (19.5)	17/102 (16.7)	31/101 (30.7)	75/349 (21.5)
*International Journal of Obstetric Anesthesia*	1.793	1/17 (5.9)	4/22 (18.2)	5/22 (22.7)	5/43 (11.6)	15/104 (14.4)
(Europe)		0/0 (0.0)	0/2 (0.0)	0/0 (0.0)	0/2 (0.0)	0/4 (0.0)
Basic Science		1/17 (5.9)	4/20 (20.0)	5/22 (22.7)	5/41 (12.2)	15/100 (15.0)
Clinical						
*European Journal of Anaesthesiology *(Europe)	1.679	2/78 (2.6)	1/122 (0.8)	3/121 (2.8)	5/129 (3.9)	11/450 (2.4)
Basic Science		1/16 (6.3)	0/21 (0.0)	0/21 (0.0)	1/24 (4.2)	2/82 (2.4)
Clinical		1/62 (1.6)	1/101 (1.0)	3/100 (3.0)	4/105 (3.8)	9/368 (2.5)
*Journal of Cardiothoracic and Vascular*	1.569	30/76 (39.5)	26/71 (36.6)	26/73 (35.6)	25/71 (35.2)	107/291 (36.8)
*Anesthesia *(US)		4/7 (57.1)	1/6 (16.7)	1/5 (20.0)	0/6 (0.0)	6/24 (25.0)
Basic Science		26/69 (37.7)	25/65 (38.5)	25/68 (36.8)	25/65 (38.5)	101/267 (37.8)
Clinical						
*Journal of Clinical Anesthesia *(US)	1.279	33/72 (45.8)	21/55 (38.2)	17/69 (24.6)	22/61 (36.1)	93/257 (36.2)
Basic Science		0/0 (0.0)	0/0 (0.0)	0/0 (0.0)	0/0 (0.0)	0/0 (0.0)
Clinical		33/72 (45.8)	21/55 (38.2)	17/69 (24.6)	22/61 (36.1)	93/257 (36.2)
*Anaesthesia and Intensive Care *(Australia/New	1.128	1/51 (2.0)	1/70 (1.4)	2/74 (2.7)	2/100 (2.0)	6/295 (2.0)
Zealand)		0/1 (0.0)	0/3 (0.0)	0/4 (0.0)	0/3 (0.0)	0/11 (0.0)
Basic Science		1/50 (2.0)	1/67 (1.5)	2/70 (2.9)	2/97 (2.1)	6/284 (2.1)
Clinical						

*Abbreviation*: IF = Journal Citation Reports^® ^Impact Factor

Significantly (P < 0.05) fewer US basic science articles were published in 2007 (97; 27.6%) and 2010 (82; 27.1%) compared with 2001 (146; 34.4%), but there were no differences in the number or percentage of US basic science published articles during 2007 and 2010 (P > 0.05; Table 2). Decreases in US basic science articles appearing in *Anesthesiology *and *Anesthesia and Analgesia *were primarily responsible for the overall decrease in US basic science articles in 2007 and 2010. A decline in US clinical research articles was also observed in 2007 (201; 15.7%) compared with 2001 (266; 19.1%) and 2004 (266; 19.3%), but a significant increase occurred in 2010 (279; 21.8%, P < 0.05 versus 2007; P > 0.05 versus 2001 and 2004). As a result of this increase in clinical research production, the percentage of all US research articles was greater in 2010 than in 2007 (22.9% and 18.3%, respectively, P < 0.05). An increase in the number of clinical research articles was observed not only in *Anesthesiology *and *Anesthesia and Analgesia*, but also in several other journals (Table 1).

**Table 2:** Cumulative basic science and clinical research publications

	2001 US/Total (%)	2004 US/Total (%)	2007 US/Total (%)	2010 US/Total (%)	Total US/Total (%)
Basic Science	146/425 (34.3)	149/439 (33.9)	97/352* (27.6)	82/303*† (27.1)	474/1519 (31.2)
Clinical	266/1392 (19.1)	266/1378 (19.3)	201/1279*† (15.7)	279/1277§ (21.8)	1012/5356 (18.9)
All	412/1817 (22.7)	415/1817 (22.8)	298/1631*† (18.3)	361/1580§ (22.9)	1486/6845 (21.7)

*Significantly (P < 0.05) different from 2001

†Significantly (P < 0.05) different from 2004

§Significantly (P < 0.05) different from 2007

The results indicate that US research contributions to the peer-reviewed anesthesia literature progressively decreased from 2001 to 2007 (22.7% to 18.3% of the totals). Declines in both basic science (34.4% to 27.6%) and clinical research (19.1% to 15.7%) articles contributed to this overall decrease. The current findings confirm the observations of Feneck *et al *who documented a significant reduction in publication rate (-2.3% per year; 95% confidence intervals of -3.4% to -1.2%) from North America (US and Canada) from 1997 to 2006 [10]. Figueredo *et al *also reported a decrease in US research production from 1997 (26.8%) to 2001 (21.8%) [11]. The number and percentage of US publications reported in this study [11] in 2001 (429 and 21.8%) were nearly identical to our findings for the same year (412 and 22.7%). Similarly, clinical research publications in 551 journals originating from US anesthesia departments decreased from 23% to 17% of totals between 2000 and 2005 [12]. These data are also very similar to those of our study in which a decrease in US clinical research articles appearing in anesthesia journals was observed from 2001 to 2007 (19.1% to 15.7%).

Szokol *et al *observed an approximately 50% reduction in the percentage of US basic science and clinical research articles published in *Anesthesiology *and *Anesthesia and Analgesia *in 2000 compared with 1980 (40.1% versus 82.2% and 38.8% versus 80.8%, respectively) [1]. A subanalysis of our data for these two journals in 2001 (percentages of US basic science and clinical research articles of 43.8% and 32.5%, respectively) revealed similar findings to those described by these authors in 2000 [1]. Our data further indicated that US basic science research production further decreased in 2007, as American investigators published only 38.5% of all articles appearing in *Anesthesiology *and *Anesthesia and Analgesia *during the year. Similar results were suggested when the 1999 data of Boldt *et al*, in which US publications were 31.2% of the world's total [13], were compared with the 2010 results of Bould and colleagues, which demonstrated that this percentage had fallen even further to 19.3% [14]. However, although the absolute number of US basic science papers continued to decrease in 2010 (89 to 78), the relative percentage increased somewhat to 44.1% in 2010 because fewer total basic science articles appeared in these two journals. In addition, a significant (P < 0.05) increase in US clinical research articles published in *Anesthesiology *and *Anesthesia and Analgesia *in 2010 was observed (41.7%) compared with 2007 (32.7%). Because American investigators often publish their results in one of these two journals, our results suggest that US research production is no longer falling and, in the case of clinical research, may actually be recovering to some extent. Indeed, the number of US clinical research publications also increased in many other anesthesia journals included in our survey, resulting in an overall increase from 15.7% in 2007 to 21.8% in 2010. This finding is potentially encouraging and suggests that a modest recovery in US anesthesia research may have begun. Alternatively, our data may indicate that US output finally reached a nadir after more than 25 years of decline (a "basement effect") and cannot decline much further as a result.

It is unclear to us whether the recent increase in US research output occurred in response to calls for action by anesthesia research leaders [5,6,8]. It is unlikely that many of the previously identified factors thought to contribute to the large decline in US research production [1,15] have been rectified, as personnel shortages, expanding clinical services, and shrinking nonclinical time continue to plague US academic anesthesia departments, thereby hampering efforts to conduct and publish research [16]. In addition, while the absolute amount of NIH funds granted to US anesthesia departments has increased in the past decade, the relative percentage of total funds allocated to anesthesia departments has remained essentially constant (< 1.0% of the NIH budget) during the ten-year duration that our survey encompassed. As a result, NIH funding for basic science and clinical anesthesia research continues to be quite limited. The NIH is the most important source of grant support for biomedical research in the US. Thus, the modest increase in US research output observed from 2007 to 2010 clearly cannot be linked to a parallel rise in funding. Instead, it may be that American anesthesiologists, despite their extensive clinical commitments, have simply begun to take more initiative as a means to enhance our collective national research profile, an approach that was recently again emphasized [17].

Our results must be interpreted within the constraints of several potential limitations. We simply counted the number of US basic science and clinical research articles, but did not conduct a formal assessment of the relative "quality" of these studies [18,19] or how often they were cited in the peer-reviewed literature. Anesthesiologists do not exclusively publish their research in anesthesia journals [12]. Thus, our analysis of 14 anesthesia journals with IF > 1 in the Web of Knowledge Journal Citation Reports^® ^"Anesthesiology" category most likely underestimated the true number of articles published by anesthesiologists to some degree. For example, some US basic science and clinical research articles were most likely published in the *Journal of Anesthesia *(a journal with an IF < 1 that is published in English), but these papers are not included in our survey because we restricted our analysis to articles published in journals with IF > 1. Many anesthesiologists are active in pain and critical care medicine research and, as a result, often publish their results in journals dedicated to these subjects. Indeed, critical care medicine, chronic pain medicine, and pain science research publications have been identified as the most highly cited papers in the field as a whole [20]. As a result, our study may have underestimated US research production because American authors may have shifted their submissions away from anesthesiology-specific to subspecialty journals. Additionally, US anesthesiology researchers have been encouraged to submit their best work to high profile journals (e.g., *New England Journal of Medicine, JAMA*) as a means to enhance the overall visibility of the specialty in the medical community [5]. Such an effort by American anesthesia researchers to publish in high impact journals may have also resulted in an underestimation of total US research output in our analysis. Nevertheless, we specifically attempted to follow the methodology of other investigators who have examined this subject to allow us to draw meaningful comparisons between our results and those of previous studies. As a result, we specifically excluded meta-analyses, even though these papers may be becoming an increasingly important part of medical research. Our analysis included only English-language publications because American researchers almost exclusively use this language. We did not discriminate between articles published by PhD researchers without clinical obligations affiliated with anesthesia departments and those by their physician colleagues. We believe that we obtained a representative sample of American anesthesia research articles in the 6,845 papers that we analyzed from 2001, 2004, 2007, and 2010, but we did not study all the articles published between 2001 and 2010 and cannot entirely exclude sampling bias as a result. Our results represent a temporal "snap shot"; it is impossible for us to speculate whether the small yet encouraging increase in US clinical research articles in 2010 is truly meaningful, will be sustained, or will be accompanied by subsequent increases in basic science research output in the near future.

## Conclusions

The current results demonstrate that US anesthesia research production progressively decreased from 2001 to 2007 as a result of declines in both basic science and clinical research. Recent basic science research output was unchanged, but a significant increase in clinical research was observed in 2010 compared with 2007. These results suggest that a modest recovery in US anesthesia clinical research may have begun.

## Methods

All data were collected in June and July 2011. Fourteen of 20 journals published in English with 2010 impact factors (IF) greater than one were chosen from the Web of Knowledge Journal Citation Reports^® ^http://www.jcrweb.com for the "Anesthesiology" subject category. When ranked using IF, these journals appeared on the first page of the Journal Citation Reports^®^, whereas those with IF less than one do not. We examined all publications in 2001, 2004, 2007, and 2010 using each journal's website. Publications were verified using the PubMed^® ^database http://www.ncbi.nlm.nih.gov. We did not include *Current Opinion in Anaesthesiology *(IF = 2.469) because this journal does not publish original research. *Minerva Anestesiologica *(IF = 2.581) was excluded because many articles in this journal were published exclusively in Italian in 2001. *Schmerz *(IF = 1.170) was also excluded because this journal is published in German. Journals dedicated solely to pain research or critical care medicine independent of anesthesia *per se *were also excluded. Basic science and clinical research articles (e.g., clinical trials, observational studies, large case series using statistical analyses) in which original data were collected for hypothesis testing were included in the subsequent analysis as previously described [10]. Editorials, review articles, special articles, small case series in which statistical analyses were not conducted, case reports, meta-analyses, audits of clinical practice patterns, abstracts, and correspondence were excluded [10]. Meta-analyses were not included to be consistent with a previous investigation [10] and because meta-analyses did not collect original empirical data. The country of origin of each article was determined and used to identify US articles and those from other parts of the world. When authors from within and outside the US contributed to an article, the corresponding author was used to establish the article's country of origin. The authors were able to reach an agreement on the definition of a basic science or clinical research article and its country of origin for all publications included in the analysis without the need for consultation with a third-party intermediary.

Pearson's Chi-square tests were used to compare categorical variables between each combination of groups of years. Linear regression analysis was used to examine the correlation between the number of US articles in each journal and the corresponding journal's IF. The null hypothesis was rejected when P < 0.05. Statistical calculations were performed using NCSS 2001 software (NCSS, Kaysville, UT, USA).

## Competing interests

The authors declare that they have no competing interests.

## Authors' contributions

PSP designed the study, collected the data, interpreted the data, wrote the original draft of the manuscript, and revised the manuscript. JAH participated in the study design, conducted the statistical analysis, interpreted the data, and edited the manuscript. Both authors read and approved the final manuscript.

## Pre-publication history

The pre-publication history for this paper can be accessed here:

http://www.biomedcentral.com/1471-2253/12/5/prepub
